# SHORT-TERM COUPLING ASSOCIATIONS BETWEEN STATE LONELINESS AND COGNITIVE PERFORMANCE IN DAILY LIFE

**DOI:** 10.1093/geroni/igae098.2101

**Published:** 2024-12-31

**Authors:** Jee eun Kang, Jennifer Graham-Engeland, Lynn Martire, David Almeida, Martin Sliwinski

**Affiliations:** The Pennsylvania State University, University Park, Pennsylvania, United States; Pennsylvania State University, University Park, Pennsylvania, United States; The Pennsylvania State University, University Park, Pennsylvania, United States; Pennsylvania State University, University Park, Pennsylvania, United States; Pennsylvania State University, University Park, Pennsylvania, United States

## Abstract

Although loneliness and neurocognitive health have been linked, little attention has been given to associations between fluctuations in state loneliness and cognitive performance. This work examines the association between within-person variation in state loneliness and cognitive performance assessed objectively in daily life, focusing on the timing and direction of the relationship. Participants from a wave of the Einstein Aging Study (EAS) were included (N=313, Mage=78 [range=70-90], 68% women, 46% White, 41% Black; average 15 years education). Over a 14-day period, participants engaged in ambulatory ecological momentary assessment burst using mobile phones, completing self-reports (including about feeling lonely at the moment) and mobile cognitive tests up to five times daily. The mobile cognitive tests assessed visual associative memory, processing speed, and spatial memory. Multilevel modeling was employed to examine day-to-day and moment-to-moment changes in loneliness and their relationship with cognitive performance, with a specific focus on concurrent, time-lagged, and reverse-lagged associations. Results revealed that higher daily loneliness negatively correlated with cognitive performance on the same day and predicted worse performance the following day, illustrating a prospective association. Within a single day, momentary loneliness negatively correlated with immediate cognitive performance, but did not predict later decreases; however, lower cognitive performance in a given moment predicted elevated loneliness later in the day. Overall, these findings highlight a complex, reciprocal relationship – loneliness predicting and being predicted by cognitive performance depending on timescale. Deepening our understanding of such temporal dynamics will be important to inform ways to break the cycle between loneliness and cognition.

